# Natural history collections as a basis for sound biodiversity assessments: Plexauridae (Octocorallia, Holaxonia) of the Naturalis CANCAP and *Tyro* Mauritania II expeditions

**DOI:** 10.3897/zookeys.870.35285

**Published:** 2019-08-06

**Authors:** Íris Sampaio, Marina Carreiro-Silva, André Freiwald, Gui Menezes, Manfred Grasshoff

**Affiliations:** 1 MARE – Marine and Environmental Sciences Centre of the Institute of Marine Research, Rua Prof. Dr. Frederico Machado 9901-862 Horta, Açores, Portugal; 2 IMAR – University of the Azores, Rua Prof. Dr. Frederico Machado 9901-862 Horta, Açores, Portugal; 3 Senckenberg am Meer, Abteilung Meeresforschung, Südstrand 40, 26382 Wilhelmshaven, Germany; 4 OKEANOS Research Unit, Faculty of Science and Technology, University of the Azores, 9901-862 Horta, Açores, Portugal; 5 University of the Azores, Rua Prof. Dr. Frederico Machado 9901-862, Horta, Açores, Portugal; 6 Senckenberg Forschungsinstitut, Senckenberganlage 25, 60325 Frankfurt am Main, Germany

**Keywords:** Alcyonacea, CANCAP project, deep water, geographical distribution, *Tyro* Mauritania II, zoological collections

## Abstract

Mapping biodiversity is the marathon of the 21^st^ Century as an answer to the present extinction crisis. A century in which science is also characterised by large scientific datasets collected through new technologies aiming to fill gaps in our knowledge of species distributions. However, most species records rely on observations that are not linked to specimens, which does not allow verification of species hypotheses by other scientists. Natural history museums form a verifiable source of biodiversity records which were made by taxonomists. Nonetheless, these museums seem to be forgotten by biologists in scientific fields other than taxonomy or systematics. Naturalis Biodiversity Center (NBC) in Leiden is care keeper of large collections of marine organisms, which were sampled in the Northeast Atlantic during the CANCAP and *Tyro* Mauritania II expeditions (1976–1988). Many octocorals were sampled and deposited in the NBC collection, where they became available for study and were partially identified by the senior author (M.G.) in the 1980s. Nonetheless, no checklist or taxonomic revision was published so far with the complete results. In 2016 the first author visited NBC to examine NE Atlantic Plexauridae octocorals. Plexauridae octocoral-vouchered records were listed and mapped to reveal high standard primary biodiversity records unreported so far for the NE Atlantic Ocean. Twenty-four Plexauridae species with ~ six putative new species to science were discovered and eleven new biogeographical records were made from distinct Macaronesian archipelagos. Finally, new depth range records were found for three species at sea basin level and for eight species at a regional scale.

## Introduction

The rate of biodiversity loss is accelerating, leading to a tendency for “Big Data” production on species observation-based occurrences instead of specimen-based occurrences as a way to map and protect biodiversity (Troudet et al. 2018). While unvouchered observations may lead to the rapid production of large datasets, specimen-based records are essential for species descriptions and for the scientific repetition principle (Cotterill 1997; Rocha et al. 2014; Troudet et al. 2018). A specimen should be available for further verification or reinterpretation, a fundamental principle of science, because properly labelled specimens are biodiversity raw data based on a researcher interpretation, from which metadata is derived (e.g., their occurrence information) (Hoeksema et al. 2012; Schilthuizen et al. 2015; Troudet et al. 2018). Moreover, vouchered records not only increase precision but are also more complete by providing ancillary data a posteriori, such as geographical positions, images or DNA sequences, which are useful for richer present and future analyses (Hoeksema 2015; Troudet et al. 2018).

Natural history museum collections (NHMC) are rich repositories representing a variety of all known life forms (Kemp 2015; Funk 2018). During 300 years of biodiversity exploration, many organisms were collected, catalogued, identified and stored under a systematic order. The name-bearing specimens deposited there are an important source of ancillary data over a long time span (Suarez and Tsutsui 2004).

The Earth’s estimated biodiversity is in the order of 10 million species, from which only 10–20% are currently known to science, while the rest still lacks a name, a description and basic knowledge on its biology (Krishtalka and Humphrey 2000; Wilson 2003; Costello et al. 2015). This lack of information on extant species is consequently reflected in an absence of understanding on which species are threatened with extinction or introduced in new environments and what is their distribution (Wilson 2003; Frey 2009; Kemp 2015). Subsequently, there is a lack of fundamental knowledge to understand the biology of species and the human-induced changes in their environment. Regarding the known biodiversity, collections-based science recognizes the value of NHMC as a source of biodiversity data in various disciplines of research (Krishtalka and Humphrey 2000; Graham et al. 2004; Costello et al. 2013; Funk 2018). NHMC species and their distribution datasets from the past, can be used to compare with present-day datasets and understand the species conservation status, prioritize and plan future studies and species management plans (Graham et al. 2004, Lister and Climate Change Research Group 2011, Funk 2018).

NHMC from remote localities or environments that are otherwise difficult to access have additional value. For example, biodiversity data collection faces higher technical challenges at distant habitats such as the deep sea, which is the Earth’s largest ecosystem. Therefore, deep sea data gathering is reflected in a few pieces of a puzzled map of discoveries. Tentative exploration of the deep sea is thought to have begun in 1521 with Fernão de Magalhães attempting to sound the Pacific Ocean between two coral islands (Murray and Hjort 1912). Yet, despite recent technological developments, it is still difficult to sample this inaccessible environment due to strong currents, rough bottoms and high costs. Therefore, the deep sea is the least surveyed marine environment although having eminent species richness (Hernández-Ávila et al. 2018).

During modern deep-sea surveys, the systematic collection of benthic marine invertebrates to characterise local fauna is usually secondary, with priority being given to long-distance transects by use of deep-sea imaging technology for species occurrence data and habitat mapping. Despite a paucity in biodiversity data, benthic marine invertebrate samples tend only to be collected as by-catch after which they are only identified at high taxonomical levels or misidentified due to the absence of taxonomists onboard (Renaud et al. 2015). At the same time, new species await an average of 21 years on shelves of NHMC for the unique eye of a trained taxonomist to be described (Fontaine et al. 2012; Costello et al. 2015, Kemp 2015). Historical benthic diversity datasets gathered at NHMC, have proven to be accurate sources of baseline data on species diversity and distribution across the Atlantic Ocean for marine benthos of the Canadian Arctic and for deep-sea reef building scleractinian coral species off the southeastern United States (Ross et al. 2012; Roy and Gagnon 2016). Based on these museum records, distribution of corals and other benthic species was confirmed and new areas for exploration were suggested (Ross et al. 2012, Roy and Gagnon 2016). However, even considering the widespread use of NHMC data, there is still much work to do in order to educate scientists about specimen importance, underutilised collections and the value of NHMC as a way to improve museum collections, support taxonomy and, most of all, the quality and reproducibility of biodiversity knowledge (Costello et al. 2013; Ward et al. 2015).

**Figure 1.**
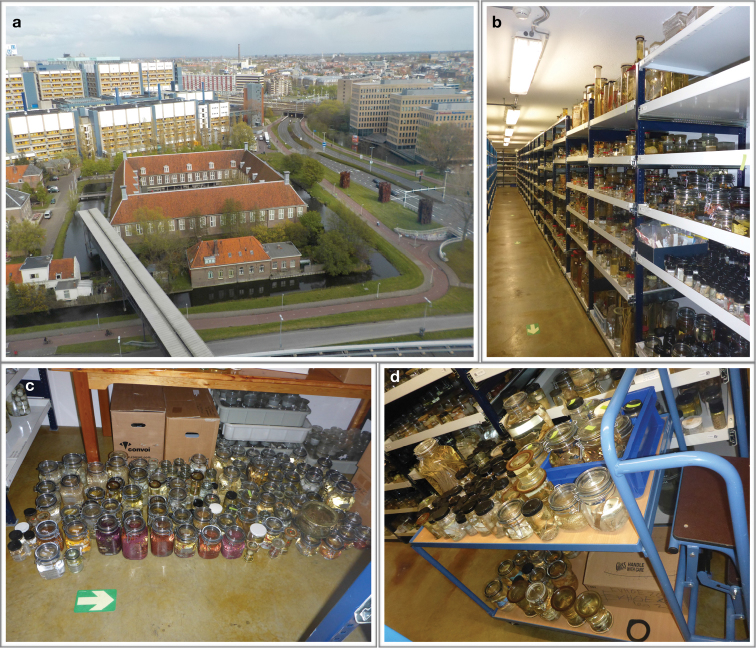
The National Museum of Natural History of The Netherlands. Naturalis Biodiversity Center in 2016 (**a**), Marine Invertebrate Collection (**b**), part of the CANCAP and *Tyro* Mauritania II consulted collection (**c, d**).

Naturalis Biodiversity Center (NBC), the national museum of natural history of the Netherlands, preserves marine benthos collected during eight explorative Dutch expeditions to the subtropical and tropical parts of the Eastern North Atlantic islands and seamounts (Figure 1). The CANCAP and *Tyro* Mauritania II expeditions took place from the Azores to Cape Verde from 1976 to 1988 on board HNLMS *Onversaagd* and HNLMS *Tydeman*, passing through all the Macaronesian archipelagos down to the west coast of Africa in Mauritania and Senegal, while sampling from the surface to 4000 m depth (Den Hartog 1984; Van der Land 1987, 1988). After the expeditions of Prince Albert I of Monaco (Thomson 1927), the CANCAP (CANarian – CAPe Verdean Deep-Sea Basin) project was the most representative set of campaigns taking place in the southern NE Atlantic Ocean with the aim of building a representative inventory and collection of organisms from 1260 sampling stations in poorly explored or unexplored regions (Den Hartog 1984). Numerous studies were published on marine benthos collected during these expeditions (see for instance Van Soest 1988; Fransen 1991; Ansín-Agís et al. 2001; Van der Linden 1998; Dijkstra and Goud 2002; Vervoort 2006), including some on octocorals: *Spinimuricea atlantica* (Johnson, 1862) from Madeira (Grasshoff 1992), the genus *Alcyonium* Linnaeus, 1758 (Stokvis and Ofwegen 2006; Sampaio et al. 2016) and some Alcyonacea of the Azores (Braga-Henriques et al. 2013). Yet, several octocorals deposited at NBC have since remained unstudied for 40 years.

Global octocoral taxonomy has been in the hand of fewer than ten scientists during the 20^th^ Century in the time of the taxonomic impediment (see Coleman 2015). This concept is based on decreased investment in taxonomy, NHMC, qualification of scientists in taxonomy and replacement or recruitment of curators and taxonomists that is associated to limited knowledge on biodiversity (Taylor 1976). With most experts currently retired, taxonomic work on octocorals is now a part-time job or hobby for some of them. The number of experts has since decreased and the interest of the new generations for this discipline is reduced or not supported. Currently, there is no curator for Octocorallia in any of the most important natural history museums of Europe. Dr. Leen P. van Ofwegen, curator of Octocorals at NBC (Leiden), was the last when he retired in 2017.

Most recent octocoral taxonomic studies in the Atlantic Ocean have focused on the northwestern Atlantic, with the northeast Atlantic receiving less attention. Within Octocorallia, the family Plexauridae Gray, 1859 is characterised by mostly arborescent colonies, branches appearing laterally, dichotomously or pinnately. Plexauridae polyps are retractile or have calyces where the anthocodiae is withdrawn and their axis has a large, hollow and cross-chambered central core encircled by gorgonin and horny loculated spaces with non-sclerite calcareous matter (Bayer 1956). Plexauridae is one of the most diverse and abundant families of octocorals, with 47 valid genera (Cordeiro et al. 2019) and several of its species known to form coral gardens (Grasshoff 1977). However, it is not the main study object of any of the current leading experts on Octocorallia taxonomy.

At the NE Atlantic Ocean, a Plexauridae species was described by Johnson (1861) for Madeira. Later, the scientific campaigns of Prince Albert I of Monaco resulted in two volumes including new plexaurid species of this area (Studer 1901; Thomson 1927), which were later revised by Carpine and Grasshoff (1985). Thomson (1929) also described some species and the French expedition Biaçores resulted in several records of Plexauridae from the Azores (Tixier-Durivault and D’Hondt 1974). In the 1970s, more species were found at the Mediterranean Sea (Carpine and Grasshoff 1975). Moreover, the last taxonomic revision of this family (still under the name Paramuriceidae Bayer, 1956) was published at the end of the decade (Grasshoff 1977). Twenty-three species in eight genera, occurring from the coast of Ireland to the Gulf of Guinea including the Macaronesian archipelagos, nearby seamounts, and the Mediterranean Sea, were accepted (Grasshoff 1977). Since this revision, little has been added in terms of biodiversity, with only one new species described, *Thesea talismani*Grasshoff 1986 (see Grasshoff 1986). Another species is also considered for Europe by the European Register of Marine Species (ERMS) (Costello et al. 2001) but this species is *Swiftia pallida*, which is a synonym of *S. dubia* (Grasshoff 1986). So far in the southern part of the NE Atlantic Ocean, 17 of the 23 valid NE Atlantic and Mediterranean Sea plexaurid species have been recorded (Grasshoff 1977; Carpine and Grasshoff 1975; Grasshoff 1986).

**Figure 2.**
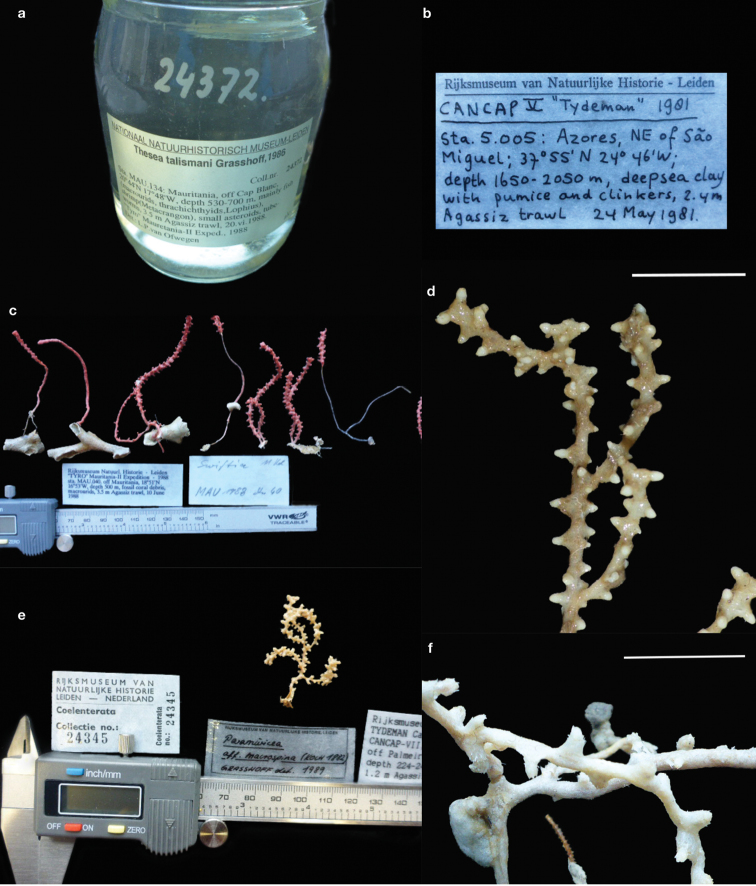
CANCAP and *Tyro* Mauritania II Plexauridae collection: **a** label of a catalogued record of *Thesea talismani* in Mauritania **b** label of previously unidentified and uncatalogued record from the Azores archipelago **c** different colonies of a new species from a coral garden filmed in situ in 2016 but stored in NBC since 1988 **d** cf. *Placogorgia* sp. I (RMNH. COEL. 42336) found mixed with specimens of the primnoid *Callogorgia verticillata* (Pallas, 1766) **e** the putative new record of *Paramuricea macrospina* in the NE Atlantic Ocean **f** detail of a new record for the Azores archipelago, *Paramuricea biscaya* (RMNH. COEL. 423339). Scale bars: 1 cm.

Plexauridae specimens collected during the CANCAP and *Tyro* Mauritania II expeditions deposited at the NBC were identified by the last author (Table 1; Figure 2). However, his work was never published, with the exception of some records (Grasshoff 1992). With the aim of documenting this valuable source of unpublished information, the Plexauridae specimens collected during these expeditions were examined by the first author: 1) to make available a list of plexaurid octocorals collected during CANCAP and *Tyro* Mauritania II expeditions; 2) to use Plexauridae records to produce maps of their geographic and depth ranges in the NE Atlantic Ocean to inform future research, field surveys and management plans; and 3) to demonstrate the value of museum records as a source of high quality biodiversity information.

**Table 1.** Plexauridae collected during CANCAP and *Tyro* Mauritania II expeditions. Geographical and bathymetrical distribution on the southern NE Atlantic archipelagos and at Mauritania including the previous data available (references) and new records (remarks). Bold script indicates new regional records.

Species	Depth Range (m)	Azores (m)	Madeira (m)	Selvagens Islands (m)	Canary Islands (m)	Cape Verde (m)	Mauritania (m)	Remarks	References
*Bebryce mollis* Philippi, 1842	71–1250	105–1250			95–330	875–900		New lower depth limit in Canary Islands (330 m).	Studer 1901; Thomson 1927; Aurivillius 1931; Stiasny 1939, 1940; Tixier-Durivault and D´Hondt 1974; Carpine and Grasshoff 1975; Grasshoff 1977, 1985a, 1989, 1992; Carpine and Grasshoff 1985; Brito and Ocaña 2004
*Muriceides lepida* Carpine & Grasshoff, 1975	79–1350	500–550	**300–400**		**180–330**	**1000–1350**		New lower depth limit for the species (1350m). Specified regional depth ranges.	Carpine and Grasshoff 1975; Grasshoff 1977, 1981, 1985
*Muriceides paucituberculata* (Marion, 1882)	51–2165	454–1350	1968		x	**515**	51	New at Cape Verde. New lower depth limit at Azores (1350m).	Studer 1901; Thomson 1927; Thomson 1929; Pax and Müller 1954; Tixier-Durivault and D´Hondt 1974; Grasshoff 1977, 1981, 1982b, 1986, 1989; Carpine and Grasshoff 1985; Brito and Ocaña 2004
*Paramuricea biscaya* Grasshoff, 1977	1094–4152	**1650–2050**		**2100–2500**	1200–1500			Specified regional depth range at the Azores and Selvagens Islands.	Grasshoff 1982a; Brito and Ocaña 2004; Molodtsova et al. 2008
*Paramuricea candida* Grasshoff, 1977	1069–1350	1069–1350						New lower depth limit for the species and at the Azores (1350m).	Tixier-Durivault and D´Hondt 1974; Grasshoff 1977, Mironov and Krylova 2006
*Paramuricea grayi* (Johnson, 1861)	20–2195		125–2195		40–600	225–1311	40–51		Johnson 1861; Thomson 1929; Carpine and Grasshoff 1985; Grasshoff 1977, 1982a, 1986, 1989, 1992; Altuna 1991; Brito and Ocaña 2004
Paramuricea aff. macrospina (Koch, 1882)	224–350					224–350			–
cf. *Paramuricea* sp. I	200					200			–
cf. *Paramuricea* sp. II	280–330					280–330			–
*Placogorgia coronata* Carpine & Grasshoff, 1975	50–2200	x	990–1000		550–1800		51	New lower and higher depth limit at the Canary Islands.	Carpine and Grasshoff 1975; Grasshoff 1977, 1981, 1985b, 1986, 1989; Brito and Ocaña 2004
Placogorgia cf. graciosa (Tixier Durivault & d’Hondt, 1974)	1100–1300					1100–1300			–
Placogorgia aff. graciosa (Tixier Durivault & d’Hondt, 1974)	1200					1200			-
*Placogorgia intermedia* (Thomson, 1927)	800–1350	800–1350						New lower depth limit for the species and at the Azores (1350m).	Pax and Müller 1954; Tixier-Durivault and D´Hondt 1974; Grasshoff 1977, 1982a, 1985b; Carpine and Grasshoff 1985; Mironov and Krylova 2006
*Placogorgia terceira* Grasshoff, 1977	170–2200	599			**200**	1311		Specified regional depth at the Canary Islands.	Carpine and Grasshoff 1985; Grasshoff 1977, 1981, 1985a, 1985b, 1992
Placogorgia aff. terceira Grasshoff, 1977	200–1350				200	214–1350			–
*Placogorgia* sp. I	590–602					590–602			–
cf. *Placogorgia* sp. II	1200					1200			–
*Spinimuricea atlantica* (Johnson, 1862)	20–875		80–84		145	875			Johnson 1862; Thomson 1927; Tixier-Durivault and D´Hondt 1974; Grasshoff 1977, 1992; Carpine and Grasshoff 1985; Brito and Ocaña 2004; Ocaña et al. 2017
*Swiftia* sp.	500						500		–
Swiftia cf. dubia (Thomson, 1929)	1320–1350	1320–1350							–
Swiftia aff. dubia (Thomson, 1929)	85						85		–
*Thesea talismani* Grasshoff, 1986	462–1090						462–1090		Grasshoff 1986, 1989; Matos-Pita et al 2016; Ramos et al. 2017
*Thesea* sp.	200					200			
*Villogorgia bebrycoides* (Koch, 1887)	56–845	105–845	x		63–400				Thomson 1927; Stiasny 1940; Tixier-Durivault and D´Hondt 1974; Grasshoff 1977, 1985a, 1992; Carpine and Grasshoff 1985; Brito and Ocaña 2004

## Materials and methods

The CANCAP and *Tyro* Mauritania II expeditions from 1976 until 1988 onboard HNLMS *Onversaagd*, HNLMS *Tydeman* and RV *Tyro* operated in the area at 14°31'–39°41'N and 08°43'–39°41'W. These expeditions used a great variety of gear like trawls, dredges and van Veen grabs for the collection of biological samples, which were subsequently deposited at the NBC (Den Hartog 1984; Figures 1, 2). The electronic database of the NBC, Bioportal, was consulted for records of the Octocorallia collection resulting from these expeditions. A visit of two weeks (17–30 April 2016) allowed the first author to locate, photograph and subsample the Plexauridae gorgonians collected during the above-mentioned campaigns.

The provenance data associated with the specimens was written on original museum specimen labels, which included more information than presented in the previously published station lists (Van der Land 1987, 1988). Information on these specimen labels, which was partially unavailable at NBC’s online catalogue (https://bioportal.naturalis.nl/), was consulted to build a reference database containing 15 data fields per museum sample (Table 2).

**Table 2.** Database structure with metadata fields from museum labels of Plexauridae collected during CANCAP and *Tyro* Mauritania II expeditions in the NE Atlantic Ocean.

**Metadat**a	**Description**
Museum Number	Museum catalogue number
Taxa	Species name
Identifier	Name of expert who identified the specimen
Expedition name	Scientific campaign in which the gorgonian was sampled
Expedition code	Scientific campaign code in which the gorgonian was sampled
Station	Station from where the gorgonian was sampled
Location	Location from where the gorgonian was sampled
Latitude	Latitude of sampling station where the gorgonian was sampled
Longitude	Longitude of sampling station where the gorgonian was sampled
Depth	Depth where the gorgonian was sampled
Substrate type	Bottom type at the location from where the gorgonian was sampled
Sampling method	Gear with which the gorgonian was sampled
Sampling date	Date in which the gorgonian was sampled
N specimens	Number of specimens covered by the catalogue number
Other notes	Other details about the specimen or sampling

Museum scientists and technicians were consulted to clarify questions regarding the metadata or to add additional information like catalogue numbers to uncatalogued specimens. Species names and taxonomy were cross-checked using World Register of Marine Species (WoRMS) Cordeiro et al. (2019) in addition to Grasshoff (1977) and Sampaio et al. (2019) to include only valid scientific names. Unidentified specimens were identified based on the revision of the family Plexauridae (Grasshoff 1977), the original descriptions of each species of Plexauridae known to occur in the NE Atlantic Ocean, and reference material from various museums.

Specimens records were organised and plotted in ArcGIS 10.6 to visualise the geographical distribution and a depth plot was prepared to visualise the vertical distribution of the gorgonians. This data was compared with previous zoogeographical and bathymetrical distribution knowledge on Plexauridae species of the NE Atlantic (e.g., Studer 1901; Thomson 1927; Tixier-Durivault and D´Hondt 1974; Carpine and Grasshoff 1975, 1985; Grasshoff 1977, 1986, 1989; Brito and Ocaña 2004). New species records were analysed by region as well as new geographical and vertical distribution records.

## Results

### Biodiversity of Plexauridae from CANCAP and *Tyro* Mauritania II

**Figure 3.**
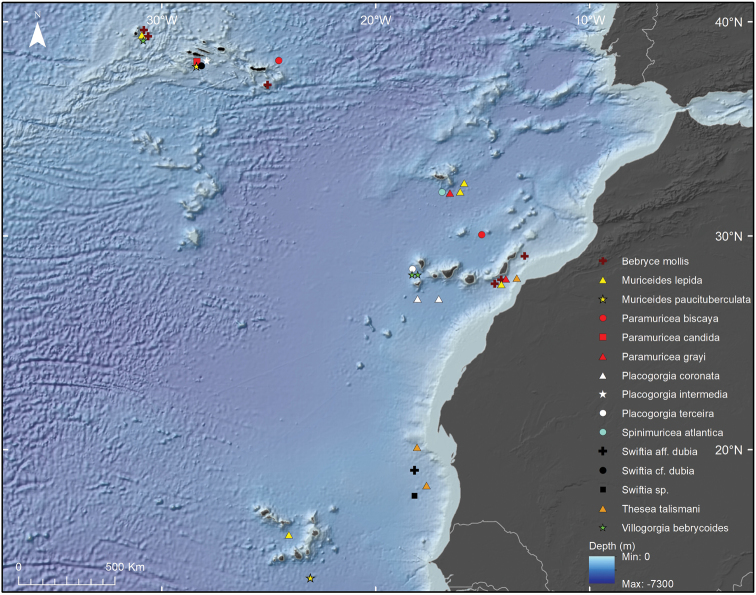
Map of Plexauridae collected during CANCAP and *Tyro* II Mauritania cruises except uncertain Cape Verdean records (see Figure 4).

**Figure 4.**
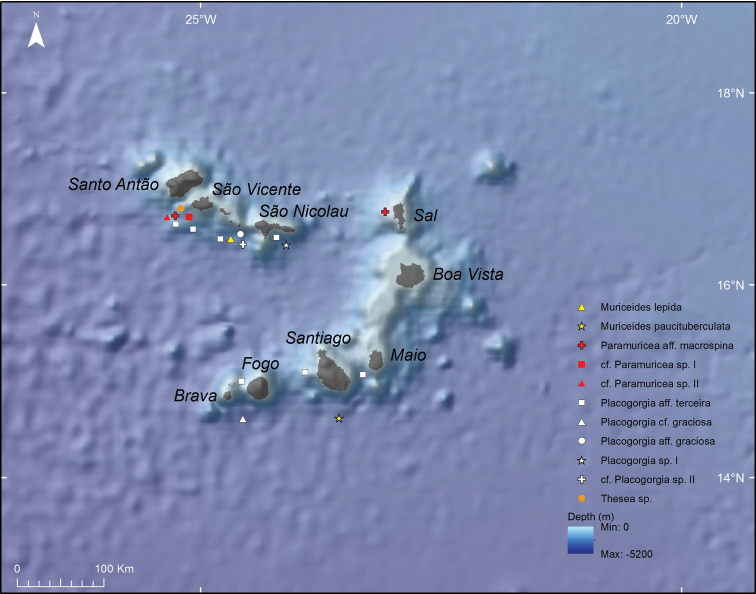
Map of Plexauridae collected during *Tydeman* Cape Verde Islands – CANCAP VI and VII cruises.

**Figure 5.**
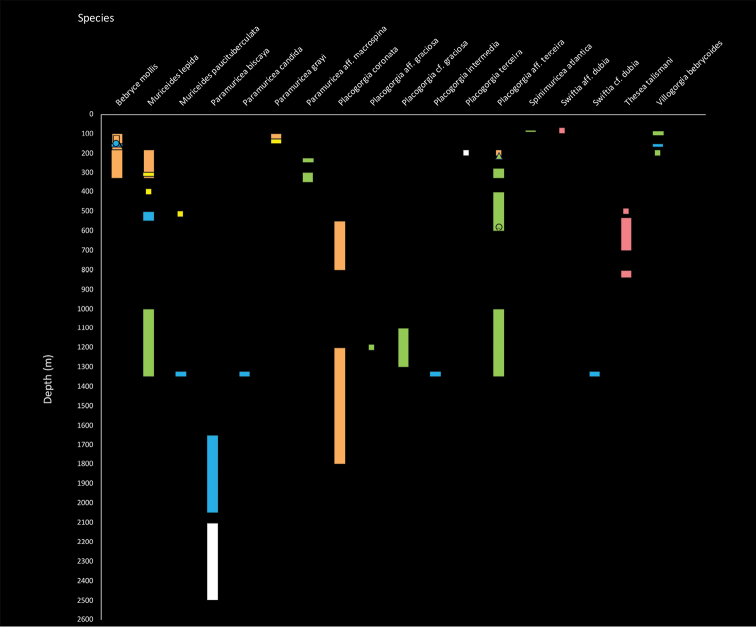
Bathymetric distribution of Plexauridae identified to species at different sampling stations of CANCAP and *Tyro* Mauritania II cruises on the NE Atlantic Ocean. Symbols represent precise records while bars represent distribution ranges. Colours represent distinct regions: Azores (blue), Madeira (yellow), Selvagens (white), Canary Islands (orange), Cape Verde (green) and Mauritania (rose).

Approximately 24 species of Plexauridae were found after studying 86 colonies, 27 fragments of gorgonians and ~24 colonies or colony fragments of gorgonians, which were sampled during the cruises of CANCAP and *Tyro* Mauritania II at the southern NE Atlantic Ocean (Tables 1, 3). The specimens were identified by the last author (M.G.) (13 species), by Dr. L.P. van Ofwegen (one species) and the first author (15 species) after the discovery of uncatalogued and unidentified specimens that were partly separated from or mixed with the catalogued species (Figures 2, 3; Table 3). Moreover, seven species were identified by two specialists (I.S. and M.G. or I.S. and L.P. van O.) (Tables 1, 3). Of the 24 plexaurid species, six lack certainty in their identification (listed as cf. or aff.) and six seem to represent species new to science (listed as sp., sp. I, and sp. II) (Tables 1, 3). The taxonomic description of these species will be presented in future works. The study produced 49 additional records of Plexauridae species that are mostly not encountered elsewhere in the NE Atlantic Ocean (Figures 2–5; Table 3).

**Table 3.** Museum records of Plexauridae octocorals collected during CANCAP and *Tyro* Mauritania II expeditions in the NE Atlantic Ocean. N corresponds to number of colonies and/or fragments. Other sampling details can be found on the list of stations published by Van der Land (1987, 1988). Bold: denotes taxonomically accurate new records, *: species identification without taxonomic accuracy and #: putative new species.

Species	Collection number	N	Identifier	Scientific campaign	Station	Location	Gear	Subtrate type
***Bebryce mollis*** Philippi, 1842	RMNH.COEL. 24337	11 fragments	Manfred Grasshoff 1989	*Tydeman* Canary Islands – CANCAP II	2.004, 28°03'N, 14°29'W, 180–330 m	Canary Islands, S of Fuerteventura, Punta de Jandia	rectangular dredge	epifauna of mixed bottom
	RMNH.COEL. 24338	6 specimens/fragments	Manfred Grasshoff 1989	*Tydeman* Canary Islands – CANCAP II	2.014, 28°03'N, 14°29'W, 200 m	Canary Islands, S of Fuerteventura, Punta de Jandia	rectangular dredge	many sponges, other epizoa
	RMNH.COEL. 24339	3 fragments	Manfred Grasshoff 1989	*Tydeman* Azores – CANCAP V	5.010, 37°41'N, 25°31'W, 150 m	Azores, S of São Miguel	van Veen grab	coarse sand, gravel, calcareous stones
	RMNH.COEL. 24340	4 specimens/fragments	Manfred Grasshoff 1989	*Tydeman* Azores – CANCAP V	5.153, 39°26'N, 31°06'W, 150–168 m	Azores, E of Flores	rectangular dredge	chama bed with fossil shells
	RMNH.COEL. 24341	7 specimens/fragments	Manfred Grasshoff 1989	*Tydeman* Azores – CANCAP V	5.166, 39°30'N, 31°06'W, 150 m	Azores, NE of Flores	rectangular dredge	shells
	RMNH.COEL. 42337	1specimen	Íris Sampaio 2018	*Tydeman* Selvagens-Canary Islands – CANCAP IV	4.096, 29°08'N, 13°25'W, 125 m	Canary Islands, E of Lanzarote	rectangular dredge	–
***Muriceides lepida*** Carpine & Grasshoff, 1975	RMNH.COEL. 24357	3 specimens	Manfred Grasshoff 1989	*Onversaagd* Madeira-Marokko – CANCAP I	1.104, 32°37'N, 16°49'W, 400 m	S of Madeira	triangular and rectangular dredge	–
	RMNH.COEL. 24358	1 specimen	Manfred Grasshoff 1989	*Tydeman* Canary Islands – CANCAP II	2.004, 28°03'N, 14°29'W, 180–330 m	Canary Islands, S of Fuerteventura, Punta de Jandia	rectangular dredge	epifauna of mixed bottom
	RMNH.COEL. 24359	1 specimen	Manfred Grasshoff 1989	*Tydeman* Madeira-Mauritania – CANCAP III	3.054, 32°43'N, 16°44'W, 300–320m	SE Madeira	rectangular dredge	–
	RMNH.COEL. 24360	2 specimens	Manfred Grasshoff 1989	*Tydeman* Azores – CANCAP V	5.187, 39°27'N, 31°05'W, 500–550 m	Azores, E of Flores	rectangular dredge	fossil corals
	RMNH.COEL. 24361	2 specimens	Manfred Grasshoff 1989	*Tydeman* Cape Verde Islands – CANCAP VI	6.096, 16°36'N, 24°39'W, 1000–1350 m	Cape Verde Islands, SW of Razo	rectangular dredge	rocky bottom with epifauna
***Muriceides paucituberculata*** (Marion, 1882)	RMNH.COEL. 24356	3 specimens, 1 fragment	Manfred Grasshoff 1989	*Tydeman* Azores – CANCAP V	5.090, 38°09'N, 28°31'W, 1320–1350 m	Azores, S of Pico	1.2 m Agassiz trawl	hard bottom with fossil corals
	RMNH.COEL. 24376	7 fragments	Manfred Grasshoff 1989	*Tydeman* Cape Verde Islands – CANCAP VII	7.026, 14°52'N, 23°33'W, 515 m	Cape Verde Islands, S of Santiago	1.2 m Agassiz trawl	–
***Paramuricea biscaya*** Grasshoff, 1977	RMNH.COEL. 24342	3 specimens	Manfred Grasshoff 1989	*Tydeman* Selvagens-Canary Islands – CANCAP IV	4.107, 30°03'N, 15°52'W, 2100–2500 m	Selvagens archipelago	2.4 m Agassiz trawl	–
	RMNH.COEL.42339	1 specimen	Íris Sampaio 2018	*Tydeman* Azores – CANCAP V	5.005, 37°55'N, 24°46'W, 1650–2050 m	Azores, NE of São Miguel	2.4 m Agassiz trawl	deep sea clay with pumice and clinkers
***Paramuricea candida*** Grasshoff, 1977	RMNH.COEL. 24343	3 specimens, a few fragments	Manfred Grasshoff 1989	*Tydeman* Azores – CANCAP V	5.090, 38°09'N, 28°31'W, 1320–1350 m	Azores, S of Pico	1.2 m Agassiz trawl	hard bottom with fossil corals
***Paramuricea grayi*** (Johnson, 1861)	RMNH.COEL. 17911	2 specimens	Manfred Grasshoff 1989	*Tydeman* Canary Islands – CANCAP II	2.047, 28°11'N, 14°02'W, 100–125 m	Canary Islands, SE of Fuerteventura, Punta de Gran Tarajal	1.2 m Agassiz trawl	mixed bottom
	RMNH.COEL.17912	1 specimen	Manfred Grasshoff 1989	*Onversaagd* Madeira-Marokko – CANCAP I	1.094, 32°39'N, 16°49'W, 125–150 m	S of Madeira	triangular dredge	mainly shells and shell agglomerates
Paramuricea aff. macrospina (Koch, 1882) *	RMNH.COEL. 24344	1 specimen	Manfred Grasshoff 1989	*Tydeman* Cape Verde Islands – CANCAP VII	7.172, 16°53'N, 25°07'W, 300–350 m	Cape Verde Islands, W of São Vicente, canal of São Vicente	rectangular dredge	small catch
	RMNH.COEL. 24345	2 specimens	Manfred Grasshoff 1989	*Tydeman* Cape Verde Islands – CANCAP VII	7.113, 16°42'N, 23°01'W, 224–248 m	Cape Verde Islands, W of Sal, off Palmeira	1.2 m Agassiz trawl	calcareous nodules
cf. *Paramuricea* sp. I #	RMNH.COEL. 42372	1 specimen	Íris Sampaio 2018	*Tydeman* Cape Verde Islands – CANCAP VII	7.171, 16°54'N, 25°06'W, 200 m	Cape Verde Islands, W of São Vicente, canal of São Vicente	rectangular dredge	no sediment, only epizoa
cf. *Paramuricea* sp. II #	RMNH.COEL. 42344	2 specimens, 3 fragments	Íris Sampaio 2018	*Tydeman* Cape Verde Islands – CANCAP VII	7.179, 16°58'N, 25°03'W, 280–330 m	Cape Verde Islands, W of São Vicente, canal of São Vicente	3.5m Agassiz trawl	sponges and soft corals
***Placogorgia coronata*** Carpine & Grasshoff, 1975	RMNH.COEL. 24347	1 specimen	Manfred Grasshoff 1989	*Tydeman* Canary Islands – CANCAP II	2.131, 27°40'N, 18°10'W, 1200–1800 m	Canary Islands, SW of Hierro, off Punta de Orchilla	1.2 m Agassiz trawl	–
	RMNH.COEL. 24348	1 specimen	Manfred Grasshoff 1989	*Tydeman* Canary Islands – CANCAP II	2.162, 27°35'N, 17°59'W, 550–800 m	Canary Islands, S of Hierro, off Punta de la Restinga	rectangular dredge	volcanic rocks
Placogorgia cf. graciosa (Tixier Durivault & d’Hondt, 1974) *	RMNH.COEL. 42341	1 specimen	Íris Sampaio 2018	*Tydeman* Cape Verde Islands – CANCAP VI	6.049, 14°52'N, 24°32”W, 1100–1300 m	Cape Verde Islands, SW of Fogo	Agassiz trawl	basaltic rocks and sandy clay
Placogorgia aff. graciosa (Tixier Durivault & d’Hondt, 1974) *	RMNH.COEL. 42342	3 specimens/fragments	Íris Sampaio 2018 (unknown identifier of the genus level)	*Tydeman* Cape Verde Islands – CANCAP VII	7.140, 16°35'N, 24°36'W, 1200 m	Cape Verde Islands, S of Razo	rectangular dredge	old lobster spot with about 500m nylon rope, with numerous epizoa
***Placogorgia intermedia*** (Thomson, 1927)	RMNH.COEL. 24349	1 specimen, 2 fragments	Manfred Grasshoff 1989	*Tydeman* Azores – CANCAP V	5.090, 38°09'N, 28°31'W, 1320–1350 m	Azores, S of Pico	1.2 m Agassiz trawl	hard bottom with fossil corals
***Placogorgia terceira*** Grasshoff, 1977	RMNH.COEL. 42369	1 specimen	Íris Sampaio 2018	*Tydeman* Selvagens-Canary Islands – CANCAP IV	4.153, 28°38'N, 17°59'W, 200 m	Canary Islands, SW of Palma	1.2m Agassiz trawl	–
Placogorgia aff. terceira Grasshoff, 1977 *	RMNH.COEL. 42370	1 specimen	Íris Sampaio 2018	*Tydeman* Selvagens-Canary Islands – CANCAP IV	4.153, 28°38'N, 17°59'W, 200 m	Canary Islands, SW of Palma	1.2 m Agassiz trawl	–
	RMNH.COEL. 24350	2 specimens	Manfred Grasshoff 1989	*Tydeman* Cape Verde Islands – CANCAP VI	6.096, 16°36'N, 24°39'W, 1000–1350 m	Cape Verde Islands, SW of Razo	rectangular dredge	rocky bottom with epifauna
	RMNH.COEL. 24351	5 specimens	Manfred Grasshoff 1989	*Tydeman* Cape Verde Islands – CANCAP VI	6.021, 15°01'N, 23°44'W, 600–400 m	Cape Verde Islands, W of São Tiago	rectangular dredge	mud and basalt rocks
	RMNH.COEL. 24352	2 specimens	Manfred Grasshoff 1989	*Tydeman* Cape Verde Islands – CANCAP VII	7.041, 14°57'N, 24°38'W, 580 m	Cape Verde Islands, E of Cima	1.2 m Agassiz trawl	gorgonians and sponges
	RMNH.COEL. 24353	1 specimen	Manfred Grasshoff 1989	*Tydeman* Cape Verde Islands – CANCAP VII	7.052, 15°06'N, 23°15'W, 594 m	Cape Verde Islands, SW of Maio	van Veen grab	practically no sediment
	RMNH.COEL. 24354	1 specimen	Manfred Grasshoff 1989	*Tydeman* Cape Verde Islands – CANCAP VII	7.136, 16°33'N, 24°17'W, 214 m	Cape Verde Islands, SE of São Nicolau, off Preguiça	rectangular dredge	calcareous nodules/algae
	RMNH.COEL. 24355	2 specimens	Manfred Grasshoff 1989	*Tydeman* Cape Verde Islands – CANCAP VII	7.174, 16°45'N, 25°07'W, 1070- 1130 m	Cape Verde Islands, SW of São Vicente	1.2 m Agassiz trawl	basaltic gravel with echinoderms
	RMNH.COEL. 42345 RMNH.COEL. 42371	1 specimen	Íris Sampaio 2018	*Tydeman* Cape Verde Islands – CANCAP VII	7.179, 16°58'N, 25°03'W, 280–330 m	Cape Verde Islands, W of São Vicente, canal of São Vicente	3.5 m Agassiz trawl	sponges and soft corals
*Placogorgia* sp. I #	RMNH.COEL. 42336	1 specimen	Íris Sampaio 2018	*Tydeman* Cape Verde Islands – CANCAP VII	7.131, 16°32'N, 24°16'W, 590- 602 m	Cape Verde Islands, SE of São Nicolau	1.2 m Agassiz trawl	muddy bottom with gorgonids and sponges
cf. *Placogorgia* sp. II #	RMNH.COEL. 42371	1 specimen	Íris Sampaio 2018 (unknown identifier of the genus level)	*Tydeman* Cape Verde Islands – CANCAP VII	7.140, 16°35'N, 24°36'W, 1200 m	Cape Verde Islands, S of Razo	rectangular dredge	old lobster spot with about 500m nylon rope, with numerous epizoa
***Spinimuricea atlantica*** (Johnson, 1862)	RMNH.COEL. 17910	Specimen not located	–	*Onversaagd* Madeira-Marokko – CANCAP I	1.092, 32°39'N, 16°50'W, 80–84 m	S of Madeira	rectangular dredge	corals (mainly dead) and shells
*Swiftia* sp. #	RMNH.COEL. 42327 RMNH.COEL. 42328 RMNH.COEL. 42329	11 specimens	Genus level: Manfred Grasshoff. Íris Sampaio is describing the new species.	*Tyro* Mauritania II	MAU 040, 18°51'N, 16°53'W, 500 m	off Mauritania	3.5 m Agassiz trawl	fossil coral debris, macrourids
Swiftia cf. dubia (Thomson, 1929)*	RMNH.COEL. 42340	3 specimens	Genus level: Manfred Grasshoff 1989; Species level: Íris Sampaio 2018	*Tydeman* Azores – CANCAP V	5.090, 38°09'N, 28°31'W, 1320–1350 m	Azores, S of Pico	1.2 m Agassiz trawl	hard bottom with fossil corals
Swiftia aff. dubia (Thomson, 1929)*	RMNH.COEL. 42374	1 specimen	Íris Sampaio 2018	*Tydeman* Madeira-Mauritania – CANCAP III	3.158, 19°22'N, 16°51'W, 85 m	off Mauritania	2.4 m Agassiz trawl	hard bottom, sponges, brown algae
***Thesea talismani*** Grasshoff, 1986	RMNH.COEL. 24371	1 specimen	L.P. van Ofwegen	*Tyro* Mauritania II	MAU 041, 18°51'N, 16°56'W, 800–840 m	off Mauritania	3.5 m Agassiz trawl	muddy bottom, tubeworms, asteroids, red shrimp
	RMNH.COEL. 24372	3 specimens	L.P. van Ofwegen	*Tyro* Mauritania II	MAU 134, 20°44'N, 17°48'W; depth 530–700 m	Mauritania, off Cap Blanc	3.5 m Agassiz trawl	mainly fish (macrourids, thrachichthyids, *Lophius*), shrimp, asteroids, tube worms
	RMNH.COEL. 42373	2 specimens	Íris Sampaio 2018	*Tydeman* Canary Islands – CANCAP II	2.058, 27°58'N, 13°24'W, 500 m	Morocco, W of Cape Yubi	5 m beam trawl	muddy bottom
*Thesea* sp. #	RMNH.COEL. 42343	4 specimens/fragments	Íris Sampaio 2018	*Tydeman* Cape Verde Islands – CANCAP VII	7.171, 16°54'N, 25°06'W, 200 m	Cape Verde Islands, W of São Vicente, canal of São Vicente	rectangular dredge	no sediment, only epizoa
***Villogorgia bebrycoides*** (Koch, 1887)	RMNH.COEL. 24370	3 specimens	Manfred Grasshoff	*Tydeman* Azores – CANCAP V	5.153, 39°26'N, 31°06'W, 150–168 m	Azores, E of Flores	rectangular dredge	chama bed with fossil shells
	RMNH.COEL. 42338	3 specimens	Íris Sampaio 2018	*Tydeman* Selvagens-Canary Islands – CANCAP IV	4.153, 28°38'N, 17°59'W, 200 m	Canary Islands, SW of Palma	1.2 m Agassiz trawl	
	RMNH.COEL. 42346	7 specimens	Íris Sampaio 2018	*Tydeman* Selvagens-Canary Islands – CANCAP IV	4.143, 28°38'N, 17°58'W, 110–86 m	Canary Islands, SW of Palma	rectangular dredge	muddy bottom with oysters

### Biogeography of Plexauridae from CANCAP and *Tyro* Mauritania II

Geographical coordinates associated with the specimens were plotted in a map of the NE Atlantic Ocean. Specimens were from all Macaronesian archipelagos, as well as from off the Mauritanian coast (Table 1; Figure 3). The Cape Verde archipelago has appeared as the region with the highest species richness (11 species in four genera) followed by the Azores archipelago where eight species of six genera were recorded, the Canary Islands (seven species in five genera), the Madeira islands (three species in three genera) and Mauritania (three species in two genera), and lastly the Selvagens Islands and Morocco (one species each) (Figures 3, 4; Table 3). The generic diversity of Plexauridae is higher in the Azores than in other NE Atlantic regions. Moreover, species identified in the Azores have a more accurate identification, especially if compared with the Cape Verde plexaurid fauna, where 11 species represent four genera but, near half of them are putative new species to science (Figures 3, 4; Table 3). The uncertainty associated with the identification of another four species from the Cape Verde islands, namely Paramuricea aff. macrospina, Placogorgia cf. graciosa, Placogorgia aff. graciosa, Placogorgia aff. terceira, may also represent new fauna (Figure 4; Tables 1, 3).

Specimens ancillary data has also revealed new species records. Some plexaurids are known to occur in most of the NE Atlantic basin; however, within it, the CANCAP records have widened their distribution ranges on a regional scale. Here we report *Muriceides lepida* Carpine & Grasshoff, 1975 in Madeira, Canary and Cape Verde archipelagos (Figures 3, 4; Table 1, 3) for the first time. In the Cape Verde archipelago, *Muriceides paucituberculata* (Marion, 1882), Paramuricea aff. macrospina (Koch, 1882), Placogorgia aff. graciosa (Tixier-Durivault and d’ Hondt, 1974), Placogorgia cf. graciosa (Tixier-Durivault and d’Hondt, 1974), and Placogorgia aff. terceira Grasshoff, 1977 are newly reported (Figures 3, 4; Tables 1, 3). Placogorgia aff. terceira was also found at the Canary Islands along with *P. terceira* (Figure 3; Tables 1, 3). Finally, *Paramuricea biscaya* Grasshoff, 1977 has its geographic distribution extended to the Selvagens Islands and the Azores (Figure 3; Tables 1, 3).

### Bathymetric distribution update of Plexauridae

In terms of bathymetric distribution, the depth range of various plexaurids is now also upgraded. Overall, most species were collected from their known bathymetrical range during CANCAP (Table 1). However, three species have increased their depth range in the NE Atlantic Ocean: *Muriceides lepida* Carpine & Grasshoff, 1975, *Paramuricea candida* Grasshoff, 1977 and *Placogorgia intermedia* (Thomson, 1927) (Tables 1, 3; Figure 5). *Muriceides lepida*, previously reported to live at 79–718 m depth (Carpine and Grasshoff 1975) is now reported at its deeper record from 1000–1350 m depth at the Cape Verde archipelago (Tables 1, 3; Figures 4, 5). *Paramuricea candida* Grasshoff, 1977 known to inhabit the range of 1069–1235 m depth (Tixier-Durivault and D´Hondt 1974; Grasshoff 1977) is now known to live down to 1350 m depth (Tables 1, 3; Figure 5). The same new high depth record of 1350 m depth is herein reported for *P. intermedia*, previously known to occur between 800–1235 m depth (Tixier-Durivault and D´Hondt 1974; Grasshoff 1977) (Tables 1, 3; Figure 5). Also, if Paramuricea aff. macrospina is in fact *P. macrospina*, this species widens its geographical distribution as it expands its greater depth limit to 350 m in the NE Atlantic Ocean (Cape Verde islands) (Tables 1, 3; Figures 4, 5). So far it is only known to occur at the Mediterranean, where it lives at 38–200 m depth (Carpine and Grasshoff 1975; Grasshoff 1977).

While the overall depth range has increased for three species, the vertical distribution range has increased or has been specified at a regional level in eight of the species identified with certainty. The exceptions are *Villogorgia bebrycoides* (Koch, 1887), *Paramuricea grayi* (Johnson, 1861), *Spinimuricea atlantica* (Johnson, 1862) and *Thesea talismani* Grasshoff, 1986 (Table 1). Considering the available data and the new records (Carpine and Grasshoff 1975; Grasshoff 1977, 1981, 1985a), we now know *M. lepida*’s specific depth ranges at the Azores, Madeira, Canary and Cape Verde archipelagos (Tables 1, 3; Figures 3, 5). In the Azorean archipelago, three species have increased their greater depth limit to 1350 m: *M. paucituberculata*, *P. candida* and *P. intermedia* (Pax and Müller 1954; Tixier-Durivault and D´Hondt 1974; Grasshoff 1977) (Tables 1, 3; Figure 5). There is a new depth record of *P. biscaya* from 1650–2050 m (Table 1, Figure 5). In Madeira is presently known that *M. lepida* occurs at 300–400 m depth and that *P. biscaya* occurs at Selvagens between 2100 and 2500 m depth (Table 1, Figure 5). At Canary Islands, *Bebryce mollis* Philippi, 1842 has a greater depth limit at 330 m (Brito and Ocaña 2004) (Tables 1, 3; Figure 5). Moreover, *P. coronata* has increased its regional depth range from 1200 m to 550–1800 m depth (Brito and Ocaña 2004) (Tables 1, 3; Figure 5). Also, *M. lepida* is now known to occur between 180 and 320 m depth and *P. terceira* at 200 m depth (Table 1, Figure 5).

In the Cape Verde archipelago, *M. lepida* has a record between 1000 and 1350 m depth and *M. paucituberculata* at 515 m depth (Tables 1, 3; Figures 4, 5). Moreover, there are many depth records at the archipelago with taxonomic uncertainty (Table 1). Lastly, at Mauritania *M. paucituberculata* is present at 51 m depth, Swiftia aff. dubia (Thomson, 1929) at 85 m depth and a new species of *Swiftia* sp. at 500 m depth (Tables 1, 3; Figure 5).

## Discussion

CANCAP and *Tyro* Mauritania II are the 20^th^ Century’s most comprehensive scientific expeditions after the earlier campaigns of Prince Albert I of Monaco in the Northeast Atlantic Ocean (Sampaio et al. 2019). By visiting NBC and examining the octocoral specimens collected during the CANCAP and *Tyro* Mauritania II expeditions, reliable and new taxonomic records were discovered, and a complete dataset was built based on the specimens and their labels in the museum collection. The value of handwritten specimen labels cannot be overstated considering the history they harbour on the collection and collector. Fortunately, most labels are still preserved with specimens or stored in special files of NHMC. While 131 specimens/fragments were photographed and subsampled for future taxonomic studies, the auxiliary data consulted from labels was compiled in a database used to map the geographic distributions and depth ranges of various Plexauridae (Table 2; Figures 2, 3, 4). Some records were already published or are available on the Bioportal database (Grasshoff 1992; Braga-Henriques et al. 2013). However, this is the first complete inventory of Plexauridae collected during the NBC campaigns.

The Plexauridae collected during these expeditions led to reference specimens of 12 species and new records of 24 species (Table 3). It also led to the discovery of ~ six putative new species in Mauritania and Cape Verde archipelago, representing an increased sampling effort applied to previously unexplored subtropical NE Atlantic areas (Van der Land 1987, 1988, Figures 3, 4). Future taxonomic investigations will clarify the taxonomic assignment of some of the specimens, e.g., Placogorgia aff. terceira, which can either represent new fauna or new records of known fauna. Provenance data stored with specimens in this NHMC have produced an extended geographical distribution range for six plexaurid species in the Cape Verde islands, three in the Canary islands, and one each in the Azores and Selvagens Islands (Figures 3, 4). Moreover, they increased the knowledge on the bathymetric distribution of three species at the NE Atlantic scale but also of eight species within each Macaronesian archipelago (Figure 5).

Reference material for comparisons with recently collected specimens in taxonomic studies, new species and new records of Plexauridae within the NE Atlantic Ocean would have not been possible without examination of the material at Leiden and assistance from museum scientists and technicians. Moreover, clarification on data incongruences and the discovery of uncatalogued records at the NHMC has provided the museum with new data to be updated and made available to scientists (e.g., *Villogorgia bebrycoides* RMNH. COEL. 42338; Table 3). Without examination of museum material, uncatalogued specimens would not have been identified, mapped and made available for future studies.

Henceforth, the present study has provided a more complete inventory of Plexauridae diversity in six regions of the NE Atlantic based on material at NBC that was collected 40 years earlier. This new knowledge will provide an important biodiversity baseline of the species occurring in the NE Atlantic, which will enable the detection of changes in species diversity and composition due to recent anthropogenic and climate change impacts.

### Biodiversity of NE Atlantic Plexauridae

The CANCAP and *Tyro* Mauritania II expeditions collected 15 species of the known Plexauridae through the southern NE Atlantic Ocean, representing 62.5 % of the 24 described species known to occur in this area (Grasshoff 1977, 1986, 1992; Table 3). Only the genus *Dentomuricea* was not represented in these samples. This taxon is known from the Great Meteor complex of seamounts and its known biotopes in the Azores that were not visited by the Dutch expeditions. Nonetheless, some species are still missing a definite taxonomic status, meaning that so far only 12 of the 15 identified species can be used as reference records for future studies. If Paramuricea aff. macrospina is indeed *P. macrospina*, the most expressive extension of the geographical species is here reported. This species was known as endemic to the Mediterranean Sea and therefore this may represent its first report in the NE Atlantic at the Cape Verde archipelago (Carpine and Grasshoff 1975; Table 2, Figure 4).

New regional records were found in distinct Macaronesian archipelagos. While the easier taxonomic assignments were found in Azorean specimens, the most difficult were found in Cape Verdean specimens (Figures 3, 4). Easier taxonomic assignments were found in Azorean specimens due to the higher deep-sea exploration and the effort in octocoral taxonomy within the region (Sampaio et al. 2019). Nonetheless, difficult taxonomic assignments were found for the Cape Verdean specimens due to the lack of knowledge on octocoral diversity of that archipelago (Figures 3, 4). The sampling and research effort of Azorean octocorals is much higher than those in other southern areas of the NE Atlantic Ocean, like Cape Verde, which explains why the octocoral fauna of those areas is mostly unknown (Pérez et al. 2016; Sampaio et al. 2019). As expected based on previous knowledge for the area, Azorean CANCAP plexaurids have a high species and genus richness and have revealed a new record for the region (Sampaio et al. 2019). *Paramuricea biscaya* has its type locality in the Gulf of Biscaya, and was further mentioned to occur in Tenerife, Canary Islands, the Mid-Atlantic Ridge and the Gulf of Mexico (Grasshoff 1977, 1985; Brito and Ocaña 2004; Molodtsova et al. 2008; Thoma 2013). Therefore, we know now that more than 50% of the Plexauridae species of the NE Atlantic inhabit the Azorean marine waters (Sampaio et al. 2019). Herein, we also report the first record of *P. biscaya* for Selvagens Islands (Table 3; Figures 2, 3). In the Canary Islands we raise the number of plexaurid octocorals from seven to ten with three new records for the archipelago according to previous data (Brito and Ocaña 2004): *Muriceides lepida*, *Placogorgia coronata* and *P. terceira* (Table 3; Figure 3).

Despite some sparse records found in the taxonomic literature (e.g., *Acanella arbuscula* (Johnson, 1862)) and a vast number of gorgonians mentioned for the area, no thorough revision of Octocorallia of Cape Verde was completed at this point (Molodtsova et al. 2008; Raddatz et al. 2011; Hansteen et al. 2014). In this study we report the highest number of species of Plexauridae collected in this archipelago, reflecting the two *Tydeman* expeditions that exclusively explored this area: Cape Verde islands CANCAP VI and CANCAP VII (Table 3; Figure 4). Moreover, two newly recorded species are reported for the archipelago with certainty, *Muriceides lepida* and *M. paucituberculata*, and three are still considered uncertain records Paramuricea aff. macrospina, Placogorgia aff. graciosa and Placogorgia cf. graciosa. Aditionally, in this archipelago, there are five species that seem to be new to science and are in need of further taxonomic investigation (Figures 2, 4; Table 3).

As undescribed marine species are commonly found in museum collections (Appeltans et al. 2012), also a sixth new species was discovered on the shelves of this NHMC (Sampaio et al., personal observation). After observing the samples and videos collected during the German expedition MSM 16/3 in 2010, the first author, in 2016, discovered that a new species of the genus *Swiftia* was preliminarily collected during the *Tyro* Mauritania II expedition in 1988 and identified until the genus level by the senior author (M.G.). Also from Mauritania, *Thesea talismani*, was reported as a new record for this location in 2016 based on the same German expedition and two Spanish expeditions Maurit-0911 and Maurit-1011 (Matos-Pita et al. unpublished data). However, it had been also sampled in 1988, identified and stored at NBC (Table 2; Figures 2a, 3).

Plexaurid species are commonly found forming coral ecosystems of high density (e.g., *Dentomuricea meteor* at the plateau of Great Meteor seamount) (Grasshoff 1977; Tempera et al. 2013). The existence of multiple colonies under a single museum collection number indicate that the colonies were collected during a single sampling event and may be present at the seafloor in high densities such as octocoral gardens. For example, 11 specimens of *Bebryce mollis* were collected at Punta de Jandia, South of Fuerteventura (Canary Islands) and 11 specimens of a new species of *Swiftia* sp. were sampled off Mauritania, potentially indicating coral garden communities dominated by these species in each area (Table 3).

### Biogeography of NE Atlantic Plexauridae

Zoogeographical regions of the North Atlantic Ocean have clustered for deep-sea Scleractinia by Cairns and Chapman (2001) and were further adapted for deep-sea corals in general and octocorals in particular (Watling et al. 2011; Braga-Henriques et al. 2013). These studies cluster species known from the Mediterranean Sea with NE Atlantic species of the Lusitanian region which are denominated as Lusitanian-Mediterranean species. Therefore, the majority of the species of this study (twelve) would be considered exclusively Lusitanian-Mediterranean (IIIA) while three, *Paramuricea biscaya*, *Paramuricea grayi* and *Spinimuricea atlantica*, would inhabit also the New England and Corner seamounts, Bermuda (IIC) (Watling et al. 2011; Table 4). *P. biscaya* is also known from the Gulf of Mexico (Thoma 2013). This would be in accordance with the trend found for the Azorean Alcyonacea, where most species are Lusitanian-Mediterranean (Braga-Henriques et al. 2013).

**Table 4.** Zoogeographical affinities of the Plexauridae species from CANCAP and *Tyro* Mauritania II expeditions. Abbreviations: I based on Grasshoff (1977); AA: amphi-Atlantic, MS: Mediterranean Sea and NEA: Northeast Atlantic; II based on Cairns and Chapman (2001) and Watling et al. (2011); IIIA: Lusitanian-Mediterranean; IIC: New England and Corner Seamounts, Bermuda.

**Species**	**Zoogeographical Affinity I**	**Zoogeographical Affinity II**
*Bebryce mollis*	NEA & MS	IIIA
*Muriceides lepida*	NEA & MS	IIIA
*Muriceides paucituberculata*	NEA	IIIA
*Paramuricea biscaya*	AA	IIIA & IIC
*Paramuricea candida*	NEA	IIIA
*Paramuricea grayi*	AA	IIIA & IIC
Paramuricea aff. macrospina	NEA & MS	IIIA
*Placogorgia coronata*	NEA & MS	IIIA
Placogorgia cf. graciosa; P. aff. graciosa	NEA	IIIA
*Placogorgia intermedia*	NEA	IIIA
*Placogorgia terceira*; *P.* aff. *Terceira*	NEA	IIIA
*Spinimuricea atlantica*	AA	IIIA
Swiftia cf. dubia; Swiftia aff. dubia	NEA & MS	IIIA
*Thesea talismani*	NEA	IIIA
*Villogorgia bebrycoides*	NEA & MS	IIIA

Nonetheless, if we consider the regions defined by Grasshoff (1977) for the Paramuriceidae (now Plexauridae) and take into account the boundary he suggested between the gorgonians of the NE Atlantic Ocean and the Mediterranean Sea, then six species of the 12 known Plexauridae species identified here are exclusively inhabitants of the NE Atlantic Ocean (Table 4). *Muriceides paucituberculata* and *Placogorgia graciosa* are widespread and live from the temperate waters in Ireland to the tropical waters of the West coast of Africa. *Placogorgia terceira* occurs on the vicinity of the Tropic of Cancer, between temperate and tropical waters of the southern NE Atlantic Ocean, while two species are uniquely known from the Azores, namely *Paramuricea candida* and *Placogorgia intermedia*. *Thesea talismani* is exclusive to the west coast of Africa (Grasshoff 1977, 1986). All the new putative species are exclusively known to inhabit the southern NE Atlantic Ocean, with *Swiftia* sp. only found in the West of Africa.

Six plexaurid species from the CANCAP records live in the “natural whole” (Ekman, 1935), the NE Atlantic Ocean (Lusitanian, Moroccan, Mauritanian and Macaronesian territories) and Mediterranean region, representing all five species mentioned by Grasshoff (1977) plus Paramuricea aff. macrospina. Here this species is mentioned as being located in the NE Atlantic Ocean; however, this record needs to be considered carefully, as explained above. Moreover, *Paramuricea biscaya*, *P. grayi*, and *Spinimuricea atlantica* are amphi-Atlantic (Watling et al. 2011; Table 4).

The distribution of marine invertebrates is highly influenced by oceanographic conditions (Ansín-Agís et al. 2001). Several oceanic currents and different climates influence the Macaronesian archipelagos (Amorim et al. 2017). The Equatorial counter-current, for example, seems to influence the distribution of scleractinian corals in the deep sea. Scleractinian corals at the Canary Islands, a warm temperate area, cluster together with corals from Cape Verde archipelago at the tropics. Additionally, Scleractinia from temperate areas like the Azores and Madeira archipelagos make up the large cluster of the Lusitanian Province (Cairns and Chapman 2001). However, NE Atlantic plexaurid octocorals seem to be more influenced by the climate. Further taxonomy and biogeographical analyses need to be done in order to make conclusive comments. Notwithstanding, the plexaurid species of the Canary Islands are also present at the Madeira and Azores archipelagos, while the new putative plexaurid octocoral species of Cape Verde indicate a distinction between species in temperate cold deep-sea waters and those at cold deep-sea tropical climates.

### Depth zonation of NE Atlantic Plexauridae

The present study has altered the known bathymetrical distribution ranges of a few plexaurid octocorals in the NE Atlantic (Figure 5). One species, Paramuricea aff. macrospina, had its depth range increased by 150 m and is now known from the upper mesophotic to the upper bathyal (40–350 m depth) (Grasshoff 1977). *Spinimuricea atlantica* (20–875 m), *Villogorgia bebrycoides* (63–845 m), *Bebryce mollis* (71–1250 m) and *Muriceides lepida* (80–1350 m), the last with its depth range increased by 650 m, inhabit the lower sublittoral down to the bathyal (Stiasny 1940; Tixier-Durivault and D´Hondt 1974; Carpine and Grasshoff 1975; Grasshoff 1977, 1985, 1992; Brito and Ocaña 2004). Species exclusively inhabiting the bathyal zone are *Thesea talismani* (462–1090 m), Placogorgia cf. graciosa (769–1300 m), which increased its depth range by 360 m, *Placogorgia intermedia* (800–1400 m), increasing its depth by 285 m, and *Paramuricea candida* with an additional 165 m (1069–1400 m depth) (Tixier-Durivault and d’Hondt 1974; Grasshoff 1977, 1985; Alvarez-Claudio 1993; Matos-Pita et al. unpublished data). Moreover, five species are eurybath having ranges from sublittoral or mesophotic depths until upper abyssal depths: *Muriceides paucituberculata* (51–2100 m), *Paramuricea grayi* (20–2195 m), *Placogorgia coronata* (50–2080 m), Swiftia aff. dubia (10–2400 m), and *Placogorgia terceira* (170–3200 m) (Grasshoff 1977, 1981, 1985, 1989; Altuna et al. 2010). Finally, the deepest plexaurid of the NE Atlantic is *Paramuricea biscaya* (1200–4152 m), an inhabitant of bathyal and abyssal depths (Brito and Ocaña 2004; Molodtsova et al. 2008).

### The importance of natural history museum collections for deep-sea research

Natural history museum collections harbour long-term biodiversity collection data. Museum data collected over time are prone to being incomplete (e.g., by lacking geographic locality information) (Soberón and Peterson 2004; Ross et al. 2012). Therefore, NHMC data are often overlooked in marine biodiversity assessments. CANCAP geo-referenced records from the 1970s and 80s have a low position accuracy in comparison with precise records sampled with modern submersibles and remote operated vehicles (ROVs) (see for example Englebert et al. 2015; Hoeksema et al. 2017). Most samples from the Dutch expeditions were collected by dredges and trawls which operated through transects, while only a single point position was recorded for each sampling location, detailing coordinates exclusively in degrees and minutes (Table 3).

While digitisation is improving museum data quality and standardisation, it is still essential to visit NHMC in order to have an accurate source of information on specific taxa (Roy and Gagnon 2016). Yet, even when having access to all the data available in NHMC, there are some problems to sort out. For example, among the octocorals deposited in NBC collected during CANCAP and *Tyro* Mauritania II expeditions, some specimens were not yet catalogued, other specimens were mixed with specimens from a different family of octocorals having the same catalogue number for different taxa and, a specimen of *Spinimuricea atlantica*, present on Bioportal, could not be located in the museum at present time.

Similar concerns can be raised by mapping published species records without checking the original record and its auxiliary information (Ross et al. 2012). Unvouchered records can be easily misidentified without proper local taxonomic knowledge or observable taxonomical characters essential to identify the species (Henry and Roberts 2013). In consequence, poor quality data can be used in studies that model distribution of species and may lead to overestimation of their putative distributions (Davies and Guinotte 2011; Bullimore et al. 2013; Henry and Roberts 2013). Unvouchered records in deep-sea ecology studies need to be identified with care, particularly if plexaurid octocorals are present. Plexaurids are extremely diverse and difficult to identify in video transects and in situ images, despite high definition and highly magnified imagery. Colony morphology is not the main distinctive taxonomic feature of most octocorals, including the Plexauridae. Consequently, some plexaurid species identification based on imagery can be easily mistaken as species of different families like Acanthogorgiidae and Gorgoniidae when the sclerome of the specimens is not analysed.

Even when a specimen is available, plexaurids have a remarkable and little studied variability of their sclerites, which hampers an easy identification and description of new species (Grasshoff 1977). Moreover, genetic markers currently used in the DNA barcoding of octocorals are not as effective as it would be desirable to discriminate at species level. Notwithstanding, currently it is important to consider genetics when describing new octocoral species and their cryptic diversity (Breedy and Guzman 2011; Reijnen et al. 2014). Museum specimens, when suitable for genetics, may be used to sequence vouchered reference DNA barcodes (Morín et al. 2019). Antique octocoral reference samples can be compared with recently collected samples like some of the CANCAP and *Tyro* Mauritania II Plexauridae, which were already sequenced with this goal.

Deep-sea exploration is expensive and constrained to specific areas of the vast, unexplored and difficult to sample deep sea. Likewise, deep-sea sampling cruises are limited to specific sampling gears and determined depth strata. Therefore, locations where well-curated deep-sea specimens are well identified and stored through decades, or even centuries, represent inestimable access to baseline knowledge on deep-sea biodiversity. NHMC with type and reference octocoral deep-sea specimens are money savers because they decrease the need of much new expensive and time-consuming fieldwork (Suarez and Tsutsui 2004). Museum records already proved to be useful for cold-water scleractinian reef building species off the southeastern United States and for Canadian Arctic marine benthos distribution based on online databases, selected publications and visits to museums (Ross et al. 2012; Roy and Gagnon 2016). Besides, CANCAP deep-sea plexaurids represent high standard accounts for this family because their identification was made by leading experts on taxonomy of gorgonians. Additionally, they increase the knowledge on Plexauridae, a neglected but important octocoral family, with many structural species which form vulnerable marine habitats. Therefore, visits to NHMC should be encouraged to greatly increase known biodiversity, to gather reference samples with credible taxonomic status and auxiliary data associated to specimens. Visits to museums are also essential to correct errors that are hampering the accessibility to this knowledge.

More value needs to be given to NHMC like the Octocorallia collection stored at NBC. Reliability on taxonomic knowledge, which is fundamental for the quality of the following biological knowledge, is dependent on NHMC (Suarez and Tsutsui 2004). Contemporary biodiversity studies rely on the 300 years of historical research deposited in museums to have accurate knowledge on species distribution and their changes under anthropogenic pressures and climate change (Roy and Gagnon 2016). This is particularly true for deep-sea invertebrates for which there is little taxonomical knowledge, especially in unexplored geographical areas. In some cases, like the Plexauridae from Cape Verde islands herein mentioned, NHMC store the unique existing data (Graham et al. 2004; Funk 2018).

Still, many countries have no or very limited funding for taxonomy, their natural history museums have limited personal to curate and investigate collections, there are shifts in the scientific focus of NHC towards molecular studies and a trend in the scientific community in publishing biodiversity studies based on unvouchered records (Kemp 2015; Troudet et al. 2018). This raises concerns on the reliability of identifications that cannot be verified (Costello et al. 2013). Moreover, taxonomic papers are published in low-citation indexed journals and NHMC are in high risk of not being preserved in the long run (Andreone et al. 2014). The consequence of a lack of taxonomical knowledge is the loss of irreplaceable sources of high-quality biodiversity data, and the proliferation of unvouchered misidentified records with poor or no auxiliary data which, in turn, results in a doubtful source of knowledge for future generations (Yesson et al. 2007; Funk 2018; Troudet et al. 2018). This trend is alarming, particularly in the light of a biodiversity extinction crisis.

A taxonomist-ecologist partnership would benefit museums and ecological studies improving long-term storage of ecological specimens and the quality and reproducibility of ecological studies (Ward et al. 2015). Therefore, collections-based research would complement field surveys in all biodiversity disciplines to achieve a more comprehensive understanding of the taxa under study and to discover biodiversity hotspots that can be considered priority for future conservation (Minton and Perez 2010).

Threats to biodiversity emphasise the need to decrease the Linnean shortfall by gathering information on known species based in specimen collection and also in describing new species, as rapidly as possible, to understand their vulnerability and to conserve them (Costello et al. 2015; Hortal et al. 2015; Ceríaco et al. 2016; Troudet et al. 2018). Henceforth, the understanding of the current state and future effects on the NE Atlantic Plexauridae relies on a complete data gathering exercise. Considering the heterogeneous octocoral taxonomy effort in the Macaronesian archipelagos and West Africa, historical literature and specimen collection data have a great value to fill in gaps in areas where biodiversity is still unknown (e.g., at greater depths) (Sampaio et al. 2019). The present study has generated accurate baseline octocoral taxonomic status records reliable for species distribution, biodiversity and conservation studies. This Plexauridae museum database will be beneficial to decide future field surveys in geographic and taxonomic unexplored areas and for a better management of deep-sea areas where plexaurid species are rare or form vulnerable marine ecosystems (VMEs).
